# Survival and Bone Remodeling in Hybrid Surface Dental Implants Placed with 3 Surgical Protocols up to 5 Years: A Retrospective Practice-Based Cohort Study

**DOI:** 10.3390/jcm14217699

**Published:** 2025-10-30

**Authors:** Hugo De Bruyn, Maria Pivovarova, Amke Rondas, Marie Scheldeman, Harrie Op de Laak, Stefan Vandeweghe

**Affiliations:** 1 Department of Periodontology and Oral Implantology, Oral Health Sciences, Faculty of Medicine and Health Sciences, Ghent University, Corneel Heymanslaan 10, 9000 Ghent, Belgium; 2 Dental School, Oral Health Sciences, Faculty of Medicine and Health Sciences, Ghent University, Corneel Heymanslaan 10, 9000 Ghent, Belgium; maria.pivovarova@ugent.be (M.P.); amke.rondas@ugent.be (A.R.); marie.scheldeman@ugent.be (M.S.); 3 Viedenta, Lambertusplein 24, 5921JJ Venlo-Blerick, The Netherlands; harrieodlaak@gmail.com; 4 Department of Reconstructive Dentistry, Oral Health Sciences, Faculty of Medicine and Health Sciences, Ghent University, Corneel Heymanslaan 10, 9000 Ghent, Belgium; stefan.vandeweghe@ugent.be

**Keywords:** dental implant, implant topography, freehanded, flapless surgery, bone level, bone loss, periimplantitis

## Abstract

**Background**: Single implants yield predictable survival and success using various treatment protocols. Innovations in design and surface texture improved survival and ensured crestal bone stability, crucial to avoiding biological complications. This study focuses on survival and peri-implant crestal bone remodeling during healing and function of single hybrid-surface implants (Machine Surface Coronal, MSc, Southern Implants Pty Ltd., Irene, South Africa), featuring a minimally rough coronal region and moderately rough body. The specific aims were firstly to compare the clinical outcome between 3 surgical protocols and secondly to assess whether the outcome is affected by macroscopic implant design. **Methods**: Clinical records of 120 consecutively placed single MSc-implants in private practice were scrutinized after 12–62 months in function. Implants were placed using one of three surgical protocols as selected by the surgeon based on clinical judgment and treatment indication: flap-healed surgery with healing abutment (HA), flapless surgery with HA, or immediate implant placement (IIP) with HA. Six different implant types, albeit with the same MSc-surface feature, were utilized, based on individual clinical indications. Radiographical crestal bone level changes over time were analyzed and effect of implant design, gender, smoking status and surgical protocol was explored. **Results**: 101 implants was available for analysis. Six implants failed prior to loading (5%); 30% in smokers versus 3.3% in non-smokers. Initial bone remodeling, due to biologic width formation, was 0.762 mm (SD 0.940) at time of loading and 0.933 mm (SD 1.057) after 2 years (*p* = 0.07). Steady state bone levels at final recall (12–62 months; mean 24) were irrespective of implant type (*p* = 0.51), surgical protocol (*p* = 0.10), gender (*p* = 0.557) or smoking habit (*p* = 0.27). 54% of the implants showed bone gain between loading and final, whereas only 3% had bone loss above 3 mm. **Conclusions**: Under daily clinical conditions, MSc-hybrid implants yield predictable clinical outcomes in line with contemporary implant systems, irrespective of implant length and diameter. A 5.9% early failure rate was found irrespective of smoking status, with no late failures. Failure rate dropped to 3.3% when smokers were excluded. Crestal bone remodeling at the time of loading, mimicking biologic width formation, as well as bone level changes over time, is indicative of a healthy peri-implant steady state irrespective of the surgical protocol.

## 1. Introduction

The replacement of missing teeth with dental implants is a routine procedure performed in daily clinical practice with an estimated 10-year survival of 96.4% (95% CI 95.2–97.5%) [1]. Over time, various modifications in implant design, surface texture (roughness), and surgical techniques have been introduced, which have led to improved predictability in nearly all treatment indications [2,3]. Single implants are a widely accepted treatment for replacing missing teeth with a 10-year survival rate of 95% [4]. They are a preferred choice when adjacent teeth are healthy, avoiding unnecessary preparation as with fixed bridges, and are more cost-effective compared to the traditional fixed three-unit dental prostheses [5].

**Figure 1:**
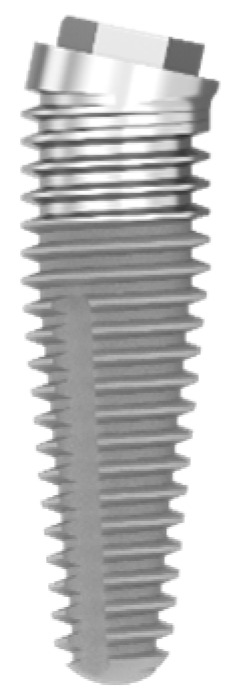
Example of hybrid MSc-IBT-12d implant (Southern Implant Inc., Irene, South Africa) as evaluated in the study; the coronal part is machined minimal rough as compared to the moderately rough apical part; the connection is external hex.

First-generation implants were machined, often coined in the literature as smooth or minimally rough. Today, most dental implants have a moderately rough surface texture, which promotes the response of bone-forming cells and enhances osseointegration [6]. Hybrid-surface implants with two distinct surface textures have been introduced, with a minimally rough surface texture in the coronal part and a moderately rough in the more apical part. An example of this implant type, as scrutinized in the current paper, is depicted in Figure 1. Systematic reviews indicate that minimally rough implants yield better peri-implant health in terms of probing pocket depths and bone level [7], but moderately rough surface implants demonstrate a lower implant failure [8]. Some studies in periodontally health patients find no difference in the clinical outcome between hybrid implants and moderately rough implants [9], but in patients with a history of severe periodontitis, minimally rough implants showed more favorable clinical parameters after 5 years of loading, when compared with moderately rough implants [10]. A significant higher crestal bone loss was observed in rough-to-the-top implants, compared to hybrid and turned implants, when placed in periodontal patients, with a follow-up of 1 to 6 years [11]. Periimplantitis is clearly linked with surface roughness as stated in a systematic review with meta-analysis; implants with minimally rough surfaces (Sa 0.5 to 1 µm) yielded a mean periimplantitis rate of 0.57% compared to 3.43% in moderately rough surfaces (Sa 1 to 2 µm) and 12.86% in rough surface implants (Sa above 2 µm) [12]. The hybrid surface implant is more and more used as the implant of first choice, and there is a trend that the industry is moving towards this surface for all kinds of indications. Often, this is poorly documented and based on in vitro studies. The suggested claims of a reduced peri-implantitis risk remain to be sustained by solid clinical research. Hence, the rationale for using a hybrid-surface implant is to improve peri-implant health without losing the advantage of optimal implant survival.

Bone resorption around dental implants is a critical factor influencing long-term survival, peri-implant health, as well as esthetic outcome. The aim of this paper is firstly to assess clinical survival and bone level changes over time of a hybrid-surface dental implant used for single tooth replacement and installed using various surgical protocols. Additionally, the study seeks to provide a deeper understanding of how factors such as implant macro-design, gender, and smoking habit may interact with bone remodeling and bone level stability over time.

## 2. Materials and Methods

### 2.1. Study Design

The study is a retrospective clinical cohort study whereby records of patients are scrutinized, and available radiographs were analyzed in order to assess bone level changes (bone loss) over time. The material includes various implant types, selected according to the treatment indication, the patient’s preference, and the condition of the recipient bone. Various surgical and loading protocols have been used without a randomized approach. Three dental master students (RA, SM, PM) have independently scrutinized the clinical records, including the available radiographs taken during surgical, prosthetic, or maintenance sessions. As the study focused on single MSc-hybrid implants, further data cleaning was performed using strict inclusion and exclusion criteria. The study protocol was agreed upon by the ethics committee of Radboud University Nijmegen, The Netherlands, as well as the regional ethics committee METC Oost-Nederland ref. 2023-16288.

### 2.2. Implant Types

All implants placed consecutively by one periodontist (DBH) in a private clinic setting (Kiesmondzorg, Dental Clinics, Panningen, The Netherlands) between July 2019 and June 2023 were logged in a practice-based register. These include various implant designs of the same implant brand (Southern Implants, Irene, South Africa), with an external hexagonal prosthetic connection and MSc-hybrid surface texture. MSc-IBNT and MSc-IBT implants are tapered hybrid implants with a diameter of 3.25 mm and 4 mm, respectively. The Machined Surface coronal (MSc) surface claims to be soft tissue friendly with reduced plaque accumulation, while the tapered body ensures effective load distribution. These implants are either straight or feature a sub-crestal angle correction of 12° or 24°, known as a Co-Axis^®^ feature. The angled design facilitates optimal prosthetic positioning in cases where the implant’s long axis does not align with the desired prosthetic outcome, such as in the anterior maxilla with buccal bone resorption. The MSc-BAT implant has a diameter of 5 mm and is characterized by its tapered design and blade-like apex, intended to enhance primary stability in compromised bone situations. The MSc-MAX implant is specifically designed for immediate molar tooth replacement and as such, the diameters range from 6 mm to 8 mm to match the socket anatomy and engage interradicular bone, providing enhanced stability [13]. The MSc-IP (Piccolo) implant has a diameter of 3 mm and is tailored for use in narrow spaces, such as the replacement of congenitally missing lateral incisors or lower central incisors where standard implant diameters are too wide.

### 2.3. Case Selection

In total, 625 MSc-surface implants of various diameters and lengths (Southern Implants, Irene, South Africa) were inserted by one surgeon (HDB) from July 2019 to June 2023. There was no exclusion based on gender, smoking, age, medical, or periodontal history. As such, the selected cohort mimics a real-world clinical practice situation. Implants supporting an overdenture (234) or a multiple implant-supported fixed prosthesis (168) were excluded in line with the focus of this paper. One hundred three single implants were excluded as they were treated and maintained by the referring dentist, resulting in data access and ethical constraints. Hence, the cohort available for radiographical assessment contained 120 single implants, surgically and prosthetically treated within one team. The crowns were either full zirconia or metal-ceramic and predominantly screw-retained.

### 2.4. Surgical Procedures

All implants (*n* = 101) were placed with a one-stage procedure, whereby the healing abutment is placed in conjunction with implant placement using three modalities: (i) in healed bone with a full thickness flap (Flap Healed + HA), (*n* = 49); (ii) freehanded non-guided flapless placement whereby the soft tissue is perforated with the drills and the alveolar crest is not exposed (Flapless + HA) (*n* = 28 implants) or (iii) immediately installed after tooth extraction with a healing abutment (Immediate Placement + HA) (*n* = 24).

### 2.5. Radiographic Assessment of Bone Loss

**Figure 2:**
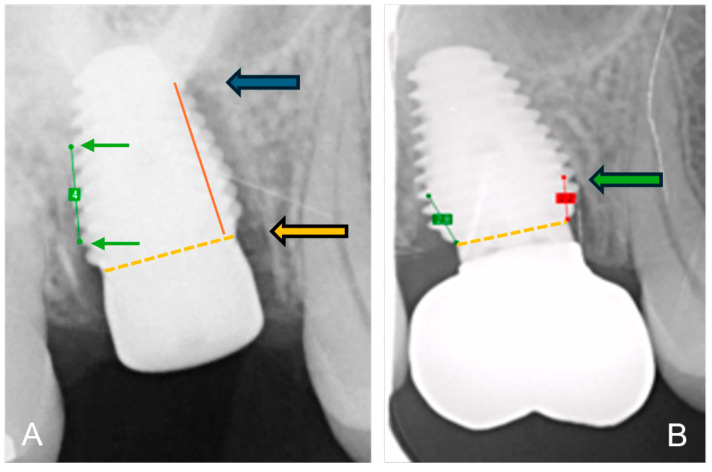
Method of radiographical assessment of an immediately placed MAX implant in a molar. Extraction socket; (**A**) after surgery: green line is the calibrated measurement of 5 implant pitch threads corresponding to 4 mm in reality; the orange dotted line indicates the implant-abutment interface and the orange arrow indicates the reference point; the red line the distance from bone level to reference point and the blue arrow indicates bone level at baseline immediately after surgery; (**B**) at final recall: the green arrow indicates the bone level at final recall; the red line the distance from bone level to reference point.

The bone-to-implant contact levels were digitally measured on digital peri-apical radiographs (Software Exquise Classic v.2024, Vertimart, Kwadijk, The Netherlands). According to the workflow applied in the clinic, periapical radiographs were taken after surgery (baseline), after loading, and at the last available recall appointment. In case the implant threads were unclear, related to misalignment of the Röntgen beam, a new radiograph was taken. The distance from the implant-abutment interface to the most coronal bone-to-implant contact was measured both mesially and distally at each of these time points. Measurement errors, due to magnification or angulation of the radiograph, were corrected by calibration with the known distance of 5 implant threads. Three master students, not involved in the actual treatment, measured the radiographs. They were calibrated, and inter-examiner and intra-examiner reliability was assessed by repeating the measurements of 24 implant cases within a 2-week interval. Bone loss at the time of loading and final recall was calculated by subtraction in relation to baseline. Figure 2 demonstrates the method of radiographical assessment of bone levels over time.

### 2.6. Statistical Analysis

The following research questions were formulated: Is the clinical outcome, being implant survival and bone level stability, in patients treated with a single hybrid dental implant: (a) depending on the surgical approach used for implant placement; (b) depending on the macroscopic implant design; (c) affected by smoking habit or (d) gender? The null-hypothesis stipulates that the outcome is independent of the afore-mentioned factors.

Statistics were obtained via SPSS version 29.0.2 (IBM Business Analytics, Amonk, New York, NY, USA). Inter-examiner reliability among the three evaluators was assessed using the Friedman test, while intra-examiner reliability was evaluated with the Wilcoxon signed-rank test to compare repeated measurements from the same examiner. The average of mesial and distal measurements resulted in one value per implant because the Wilcoxon signed-rank test showed no significant difference between them at baseline (*p* = 0.317), at loading (*p* = 0.355), and at recall (*p* = 0.453). The Kruskal–Wallis test, with appropriate Bonferroni corrections for multiple comparisons, was used to compare differences in medians across three or more groups for bone loss by implant type, loading time, surgical protocol, gender, and smoking status. Demographics are presented using descriptive statistics, and a paired *T*-test was used for bone loss comparisons between surgical groups over time. The significance level was always set at *p* < 0.05.

## 3. Results

The research cohort contained 120 single MSc-hybrid implants treated by one surgeon (HDB) and prosthodontist (HO) in the same clinic; 11 implants with incomplete follow-up and missing radiographs were dropouts (8.5%); an additional 8 were immediately loaded and did not fall in the scope of the study (6.7%). In total, 101 single implants were included for further assessment; 41 in the mandible and 60 in the maxilla; 52 were placed in molar sites, 31 in premolars, and 18 in the anterior region. Moreover, 46 were placed in females and 55 in males (median age 59 years; range 19–82).

A total of 6 single implants failed prior to loading (5.9%), but only 3/91 implants in smokers failed (3.3%) as compared to 3/10 implants placed in smokers (30%). Hence, 95 implants were included in the radiographic analysis. Details of the implant specifications are given in Table 1, Table 2 and Table 3. Implant distribution across the three surgical protocols is given in Table 4.

There was no significant difference between the three examiners regarding the repeated measurements (*p* = 0.405 for mesial and *p* = 0.131 for distal measurements); similarly, no differences were found in repeated measurements for the three examiners (Wilcoxon *p* = 0.421, *p* = 0.0307, and *p* = 0.091, respectively).

**Figure 3:**
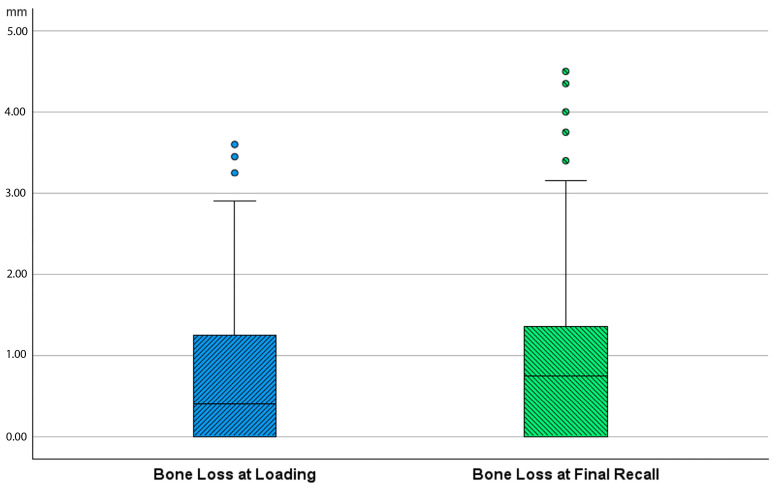
Boxplot showing bone loss in mm at time of loading and final recall as compared to baseline (after surgery) for the total cohort.

**Figure 4:**
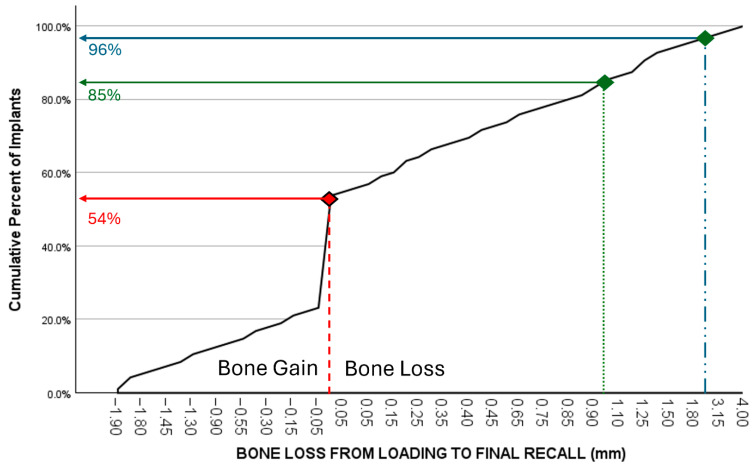
Cumulative representation of implants (*y*-axis) in relation to additional bone loss (mm) between loading and final recall (*x*-axis). 54% the implants showed additional bone gain, 85% of all implants had a bone gain or a maximum bone loss up to 1 mm, while 96% had less than 2 mm bone loss.

**Figure 5:**
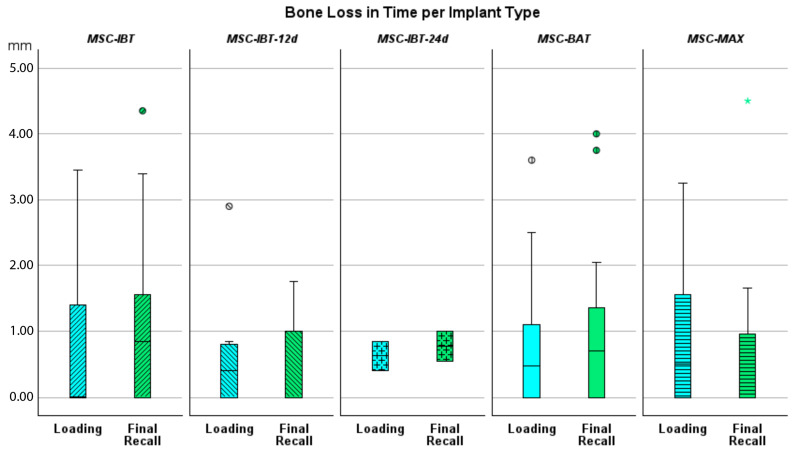
Boxplot showing bone loss in mm at time of loading and final recall compared to baseline for the various implant designs.

**Figure 6:**
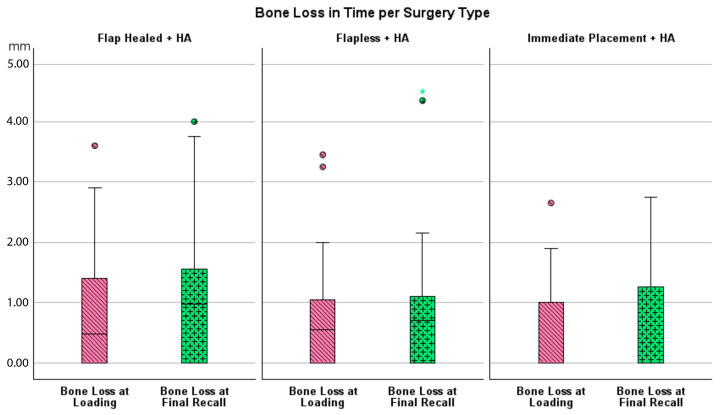
Boxplot showing bone loss at loading and final recall compared to baseline per type of surgery.

Bone loss at the time of loading and final recall for the three surgical modalities, as well as the total cohort, is expressed in Table 4. Given that all implants were inserted with a one-stage surgery, the bone loss captures the total bone remodeling (initial bone remodeling due to surgical trauma and biologic width formation, as well as possible bone loss during loading). This total mean bone loss was 0.74 mm at loading and 0.93 mm at final recall and indicative of steady state bone levels during loading (*p* = 0.068). Figure 3 represents the bone loss for the total cohort at loading and final recall. The cumulative representation of all implants and their respective bone loss between loading and final recall is visualized in Figure 4. 54% the implants showed additional bone gain, 31% of all implants had bone loss up to 1 mm, whereas only 4% of the implants had bone loss above 2 mm. Mann–Whitney U test showed no significant difference in bone loss at the final recall for gender (*p* = 0.557) nor for smoking habits (*p* = 0.273). The independent Kruskal–Wallis test showed that the distribution of bone loss at loading and final recall was the same across implant types (*p* = 0.994 and *p* = 0.510) (Figure 5) or type of surgery (*p* = 0.544 and *p* = 0.1) (Figure 6). No statistically significant difference in bone loss after loading was observed among the three surgical approaches (*p* = 0.603) (Table 4 and Figure 6). Hence, the study failed to reject the formulated null hypothesis.

**Figure 7:**
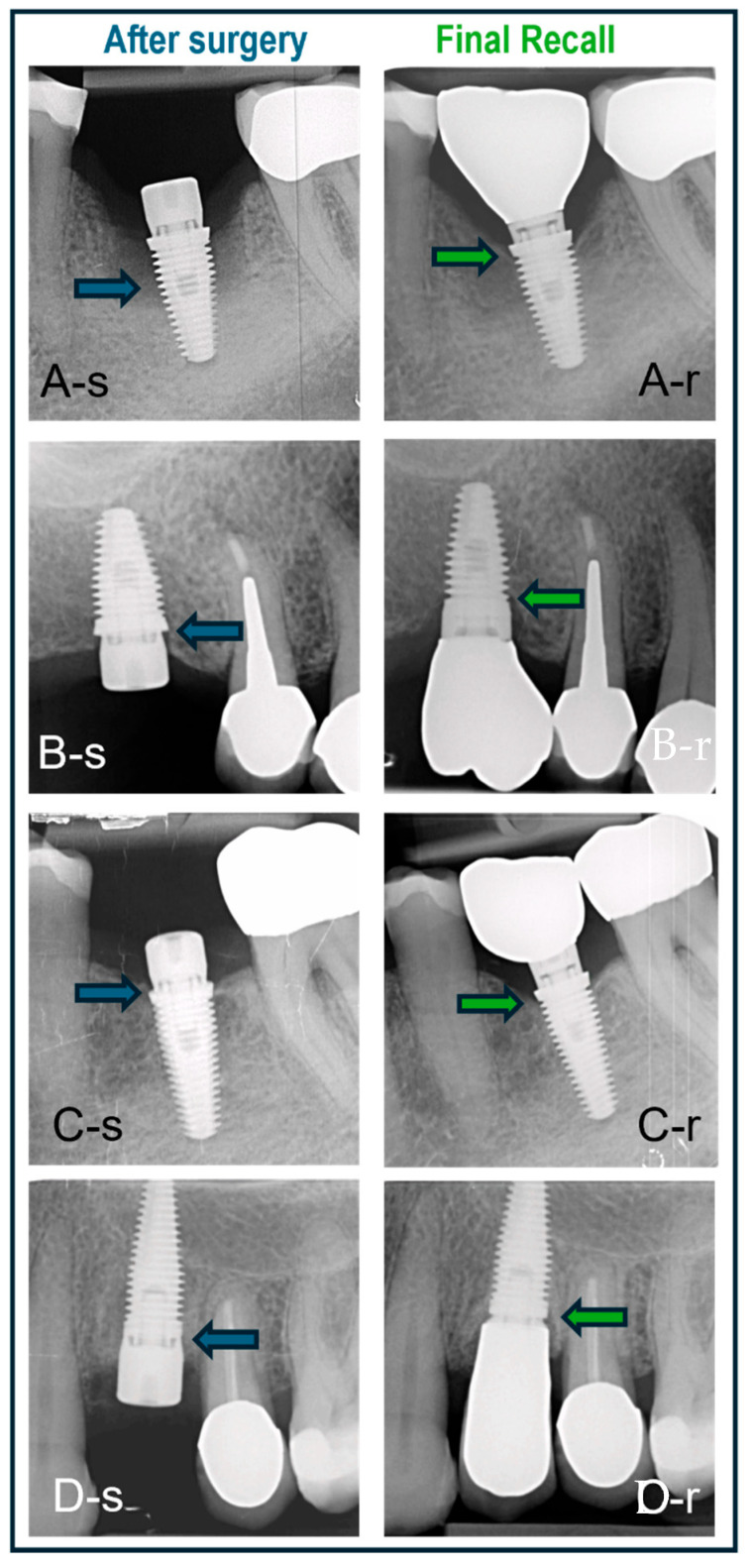
Cases treated with various surgical protocols with baseline (-s) and final recall (-r) radiographs. The blue and green arrows indicate bone level at baseline and recall, respectively. (**A**) Immediate placement of a lower molar using a BAT implant placed in an intact socket without guided bone regeneration; bone regrowth and bone maturation in the socket are evident. (**B**) BAT implant placed flaplessly in healed bone followed by minimal bone remodeling; non-platform switched crown. (**C**) BAT implant with open flap followed by installation of platform-switched crown and minimal bone remodeling. (**D**) IBT implant placed in healed bone and restored with non-platform-switched crown and no signs of bone loss.

Some clinical cases representing the three surgical protocols with different implants are shown in Figure 7.

## 4. Discussion

This retrospective clinical cohort study investigated the clinical outcome of 120 single implants and is, to our knowledge, the first to evaluate the clinical outcomes of MSc implants in a consecutively treated non-selected patient population. It provides insight into the implant performance in a routine clinical practice, including high-risk patients such as smokers or individuals with certain medical issues. The focus was on implant survival and crestal bone remodeling, and the impact of various surgical treatment protocols on the clinical outcome.

The overall failure of 3.3% in non-smokers is in line with current evidence [4]. However, when including smokers, failures increased to 5%. Smoking is widely recognized as a critical risk factor in both oral and peri-implant health, with extensive evidence linking it to reduced implant survival in both the short and long term [14] and greater marginal bone loss over time [15]. However, the study included too few smokers to draw unequivocal conclusions related to survival and bone stability. In the current study, all failures occurred prior to loading, and failed implants have been replaced successfully without further losses during function. In patients with proper oral hygiene measures and regular professional maintenance, which was the case in the assessed cohort of this study, the predictability of long-term survival is more guaranteed [16].

The single implants were installed using three possible surgical procedures. The choice was purely in the hands of the surgeon. The study did not advocate a randomized controlled approach because it was the decision of the surgeon and the patient to make a choice. In terms of implant survival, flapless surgery yielded the best results with 96.4% survival as compared to 93.9% for the flap surgery, albeit not statistically significantly different. Hence, the null hypothesis was not rejected. This clinical difference may be due to selection bias. The flaplessly treated sites had a good bone volume to prepare the implant recipient site with a freehanded, flapless approach. In cases of doubt, the surgeon opted for a flap in order to visualize the alveolar crest after raising a full-thickness flap. A similar approach was used previously by the same surgeon and yielded equal results for freehanded flapless versus flap surgery in single implants [17].

Similarly, the lowest survival of 91.7% was noted in the immediately placed implants. This is in line with a systematic review showing that implants fail slightly higher in immediately placed implants, between 90 and 95%, as compared to more than 95% survival with delayed implants placed in healed bone [18]. In this review, implants of non-hybrid design were included, suggesting that the failures are more surgically determined than related to the surface texture. It is obvious that immediate placement of an implant in an extraction socket is a more challenging approach as compared to implant placement in healed bone, although patients’ preferences should be taken into account.

Crestal bone loss around implants occurs during initial healing as a consequence of a physiological process rather than a sign of disease [19]. Furthermore, peri-implant marginal bone loss does not necessarily represent a condition of disease since reduction in marginal bone levels may be observed in a majority of patients during follow-up time with a steady state bone level after 3 to 6 months. Only a minority of those patients lose implants or implant-supported prostheses in the long term [20]. In our study, bone levels remain stable once the biologic width has been established, with no significant differences between the time of loading and final recall. All implants in the present study were installed using a one-stage surgery. Consequently, the healing abutment pierces the soft tissue from the day of surgery, and hence, initial bone remodeling starts from baseline. The periapical radiographs taken at the time of loading, therefore, reflect the initial bone remodeling, which may be an overestimation compared to most clinical studies using the radiograph of the moment of loading as baseline. Nevertheless, the MSc implants yield an initial bone loss of less than 1 mm on average with no further significant increase afterwards. However, based on consensus within the European Association for Osseointegration, mean peri-implant bone loss is not an adequate outcome to study the prevalence of peri-implantitis, while the reporting of frequency distributions of sites with bone loss exceeding internationally accepted thresholds is considered more appropriate. This threshold is currently agreed to be 3 mm from the coronal portion of the intraosseous component of the implant, with the baseline radiograph taken at the time of loading and not at the time of surgery [21]. Figure 4 represents the cumulative number of implants and their respective bone loss between loading and final recall; 54% the implants showed bone gain, and only 4% of the implants had bone loss above 2 mm. Reasons for post-loading bone loss can be multifactorial and often related to the design of crowns, oral hygiene measures, or loading. A disadvantage of the present study is that the peri-implantitis prevalence was not measured because the pocket charts were missing in the files or were questionable. Most patients returned to their own oral hygienist, who took care of individual professional maintenance. Furthermore, there is debate on the diagnostic validity of probing depths and bleeding on probing, which has commonly been used to define periodontal diseases, but may not have the same diagnostic value to assess peri-implant tissue health and disease [22]. For these reasons, we focus on bone level stability as a clinical applicable diagnostic parameter. Applying the bone level criterion as a surrogate measurement for periimplantitis risk, only 3/96 (3.1%) implants fall within the risk zone, which is an indication of the low risk for peri-implant health issues. This may be a consequence of the minimally rough coronal implant surface as confirmed in a systematic review [12].

In this study, no statistically significant differences in marginal bone loss following loading were observed among the three surgical approaches evaluated. This observation aligns with the previous literature, indicating that there is generally no significant difference in bone loss between immediate and delayed implant placement. However, delayed implant placement is often more feasible in clinical practice than immediate placement, as this approach requires careful case selection and should be performed by experienced clinicians to minimize the potential complications [22]. Additionally, while the bone levels of both techniques are comparable, some studies suggest that delayed implant placement may have a slightly higher survival rate (>95%) compared to immediate implant placement (90–95%), which is also confirmed in the present study [18]. This variation in survival rates highlights the importance of considering patient-specific factors, surgical expertise, and clinical circumstances when selecting the optimal timing for implant placement. Flapless surgery is a minimally invasive and effective option for single tooth restorations when conditions like favorable bone quality, careful patient selection, and sufficient clinician experience are met [23]. The results of this study are consistent with previous research, showing no statistically significant differences in bone levels between the two techniques at baseline, loading, or recall time points. This suggests that the choice of surgical approach has a limited impact on bone levels in the short to medium term, with other factors like implant design, loading protocols, and patient characteristics playing a more influential role in outcomes. Despite its benefits, flapless surgery has some limitations. The lack of bone visibility increases the risk of complications like dehiscence or fenestration, though none were found in these studies [23]. Additionally, determining the vertical implant position can be more difficult without bone exposure, especially in patients with narrow ridges or complex anatomy [22,24]. Overall, flapless surgery offers less discomfort and faster recovery without compromising long-term success, but careful planning and experienced clinicians are crucial for optimal outcomes [25,26,27,28]. A drawback of the present study lies in the fact that the initial bone conditions between surgical techniques may have influenced outcomes. In the flapless approach, freehanded and non-guided implant placement is performed only under optimal bone conditions, which is not necessarily the case for the Flap + HA approach. As a result, implants placed using the flapless approach may benefit from more favorable initial conditions, potentially influencing the overall prognosis. On the other hand, the presurgical selection was solely based on clinical judgment without three-dimensional radiographs like CT or CBCT. Obviously, for single tooth replacements in the vicinity of neighboring teeth, the use of these techniques bears bigger health risks in terms of radiation as well as higher treatment costs without providing additional benefits. Our study underlines the importance of clinical experience as well as the liberty of the surgeon to opt for the best feasible surgical approach or implant choice, based on appraisal of clinical conditions and related risks. It is important to consider the potential long-term implications of each approach. Some studies indicate that flapless surgery may result in less soft tissue trauma and better preservation of the bone, although this could be more evident in cases of immediate implant placement [29]. In the current study, immediate implant placement was performed in 23.8% of single implants. While this approach offers advantages such as low morbidity and even immediate provisionalization (excluded in this study), it also carries a higher risk of complications, including implant failure and infection [28,29].

The implant cohort contained various types of implants, albeit with the same surface texture (Figure 7). Despite these variations in implant design, no statistically significant differences were observed in the bone level outcomes. Hence, the null hypothesis was not rejected. The existing literature comparing hybrid surface implants with moderately rough surface implants suggests that hybrid surfaces are a viable alternative, demonstrating comparable clinical, biomechanical, and radiographic performance [30]. Additionally, platform switching was applied to certain implants. According to previous research, platform switching can potentially reduce crestal bone loss by repositioning the implant–abutment junction toward the central axis of the implant, thereby minimizing the impact of mechanical and microbial factors on the crestal bone [31]. Platform switching was found to contribute to the preservation of crestal bone. It helps maintain bone level with minimal remodeling in thick soft tissues, while in thin tissues, it may not prevent bone loss [32]. Platform-switching implants, particularly when long abutments are used, show less marginal bone loss after 6 months and 1 year [33]. The practice-based study design allowed inclusion of a large sample treated in a daily clinic setting, which is often problematic in academic studies. Drawbacks and limitations of the study are related to the retrospective and non-randomized design, and hence, are missing a power calculation. The radiographs are not standardized, which may account for measurement errors when comparing different time points. Furthermore, only one surgeon was involved in the procedures, which may also induce a selection bias, and the peri-implant soft tissue condition was non-retrievable from the patients’ records. And last but not least, the study only focused on single implants. Although the MSc surface implants have shown a predictable short-term outcome, further prospective research with more controlled studies involving multiple teams and multiple indications is suggested. These should not only focus on long-term implant survival and bone stability but also include clinical peri-implant health parameters, as well as immunomarkers or bacterial profiling. The claims made by industry that the smooth surface affects bacterial adhesion in vivo and consequently results in a reduced peri-implantitis risk should be proven clinically for a wide range of indications, among others, at-risk patients.

## 5. Conclusions

Within the limitations of this retrospective, non-randomized, and practice-based analysis, it can be concluded that the MSc surface implants, regardless of their design and surgical technique, yield a predictable outcome in terms of single-implant survival. Both clinical survival as well as bone remodeling are according to international standards and remain stable over time. Less than 3% of the implants have unacceptable additional bone loss during function.

## Figures and Tables

**Table 1:** MSc-hybrid implant diameter and related failures.

	Installed	Failed
3 mm	1	0
3.25 mm	4	0
4.0 mm	47	4
5.0 mm	37	1
6.0 mm	7	1
7.0 mm	5	0
*Total*	*101*	*6*

**Table 2:** MSc-hybrid implant length and related failures.

	Installed	Failed
6.0 mm	4	0
7.0 mm	5	0
8.5 mm	18	1
9.0 mm	2	0
10.0 mm	27	0
11.5 mm	31	1
13.0	13	4
15.0	1	0
*Total*	*101*	*6*

**Table 3:** MSc-hybrid implants with various designs and related failures.

	Installed	Failed	Survival %
IBT	40	5	87.5
IBT-COAXIS	11	0	100
BAT	38	1	97.4
MAX	11	0	100
PICOLLO	1	0	100
*Total*	*101*	*6*	*5.9%*

**Table 4:** MSc-hybrid implant survival and bone loss for various surgeries at loading and final recall; mean (SD) and statistical significance using paired *T*-Test.

	Installed	Failed (Survival %)	B Loss Loading Mean (SD)	B Loss Final Recall Mean (SD; Range)	*p*-Value (Paired *T*-Test)
Flap + HA	49	3 (93.9)	0.80 (0.94)	1.09 (1.04)	0.098
Flapless + HA	28	1 (96.4)	0.78 (0.98)	0.93 (1.19)	0.410
Immediate placement	24	2 (91.7)	0.56 (0.80)	0.60 (0.87)	0.809
Dropouts	11	-			
Excluded	8	-			
*Total*	*120*	*6 (95.0)*	*0.74 (0.92)*	*0.93 (1.06)*	*0.068*

## Data Availability

Data supporting reported results analyzed or generated during the study can, with reasonable motivation, be collected from the principal author (hugo@debruyn.one).
